# Commentary: Variability of Practice, Information Processing, and Decision Making—How Much Do We Know?

**DOI:** 10.3389/fpsyg.2021.685749

**Published:** 2021-08-04

**Authors:** Taewon Kim, David L. Wright, Wuwei Feng

**Affiliations:** ^1^Department of Neurology, Duke University School of Medicine, Durham, NC, United States; ^2^Non-Invasive Brain Stimulation Laboratory, Division of Kinesiology, Texas A&M University, College Station, TX, United States

**Keywords:** contextual interference, motor learning, practice conditions, memory consolidation, practice variability, random practice

## Introduction

The review by Czyz (2021) should be applauded for revisiting the role of practice variability for skill acquisition especially given ongoing interest in the identification of “best-practices” in instructional and therapeutic contexts as well as the need for the development of a more mechanistic framework within which memory for skill is viewed. The present commentary reviews work (Shea and Kohl, 1990, 1991), absent in the target article, which was designed to uncover how variability rather than specificity bestows a skill learning advantage. The initial theorizing of Shea and Kohl (1990, 1991), borrowed from studies targeting the contextual interference (CI) effect, is expanded to consider recent behavioral and physiological changes associated with superior skill memory resulting from experiencing practice variability in an interleaved format. We conclude that variable experiences *scheduled* in this format facilitate learning but remain more cautious in regards to the more general claim that practice variability advances skill acquisition.

## Shea and Kohl's Contribution to the Variability of Practice Debate

Shea and Kohl (1990, 1991) attempted to shed light on the efficacy of practice variability not for skill generalizability, overtly addressed by schema theory (Schmidt, 1975) and noted by Czyz (2021), but for skill retention for which schema theory was ambivalent and to which less experimental rigor has been exerted. This involved examining the delayed retention of an isometric force production skill following repeated reproduction of only a single target force (i.e., specific practice) compared to that achieved after practice of this target skill embedded in practice with other similar forces (i.e., variable practice) (Shea and Kohl, 1990). The variable practice condition led to significantly less error for executing the target force 24-h after practice. To eliminate the possibility that the observed test benefit wasn't from the insertion of the practice variations, merely resulting in greater practice extent, a second experiment included an additional condition that replaced trials with variations of the target task with an equivalent number of additional trials with the target force. Despite extra practice with the target task, learning wasn't improved. Thus, the key outcome remained the same, exposure to variations in force production was important to secure successful retention of the target force.

These data then extended the efficacy of practice variability beyond skill generalizability to include skill retention (see Czyz, 2021). Shea and Kohl (1990) entertained two potential explanations for their finding. First, the inclusion of practice of task variations merely acts to create a temporal divide between repetitions of the target skill that increased the likelihood that a complete set of planning operations was conducted on each trial, especially for the target task. Alternatively, executing task variations of the target skill provides a unique opportunity to complete cross-comparative processes with the criterion task which fosters a more detailed memory for the target skill being established. Shea and Kohl (1991) designed a separate set of experiments to determine which of these explanations best accounted for the retention benefit of variable practice. In the first experiment, execution of a tracking task was embedded with trials of the target force production skill used in Shea and Kohl (1990). The prediction was that if variable experiences merely serve to create forgetting to induce more elaborate planning on each trial of the target skill, then performing the tracking task should also induce forgetting which in turn should still improve retention. On the other hand, if variable experiences are critical to the development of novel skill memory rather than just inducing forgetting, practice with the tracking skill should be less effective for learning than the variable practice condition. The latter outcome was observed by (Shea and Kohl, 1991, Experiment 2) verifying that practice of skill variations, in conjunction with the target skill, makes a unique contribution to skill memory development. This claim was further supported in a second experiment that revealed that increasing the number of task variations (i.e., 3 v 1) that were practiced between repetitions of the target skill, that is, systematically introducing greater variability, was associated with further improvement in skill retention.

## Explaining shea and Kohl's Findings: Then and Now

Similar to Czyz (2021) in the target article, Shea and Kohl (1990, 1991) initial theorizing as to how practice variability supports skill memory was borrowed from the initial formulations of the *forgetting-reconstruction* (Lee and Magill, 1983, 1985) and the *elaboration* (Shea and Zimny, 1983) accounts for contextual interference (CI) effect. The CI phenomenon addresses a learning advantage afforded by using interleaved practice (IP) compared to repetitive practice (RP) when acquiring multiple skills concurrently. The forgetting-reconstruction position (Lee and Magill, 1983, 1985) proposes that the extensiveness of trial-to-trial processing is relatively greater during IP as a result of the frequent interchange of task information that must occur in working memory throughout IP. In contrast, RP involves executing the same response repeatedly for a pre-determined number of trials before experience with new tasks demands thus reducing the need to reconstruct an action plan as practice continues. The reduction in preparatory activity necessitated by RP is assumed to hinder subsequent retention efforts.

The elaboration perspective argues that IP offers the opportunity to develop a more elaborate description of a novel skill memory by encouraging extensive use of inter-task processing throughout training. The concurrent presence of information about multiple skill in working memory affords the assessment of similarities and differences between the skills being acquired. Engagement of inter-task processing is argued to be critical to forming a robust and intricate memory network that supports access to task-specific knowledge at a later time.

While Shea and Kohl (1990, 1991) interpretation of their data leaned heavily in favor of the elaboration account, more recent evaluations of the underlying reasons for the efficacy of an interleaved presentation of practice variability includes features of both positions. For example, both the forgetting-reconstruction and elaboration explanations predict relatively greater attention demands during IP which has been verified as greater dual-task cost during this practice format (Li and Wright, 2000). At the behavioral level, the additional planning cost during IP is due, at least in part, to more challenging response programming linked to the development of more resilient motor chunks (Immink and Wright, 2001; Wright et al., 2005). There is also evidence that greater decision making or response selection demands occur during IP as alluded to in the target article (Czyz, 2021).

Consistent with large attention demands associated with IP is the report of greater investment in task-related planning and/or execution operations during IP in numerous neural imaging studies (Cross et al., 2007; Wymbs and Grafton, 2009; Lin et al., 2011, 2013). Specifically, individuals faced with IP exhibit a broader activation of neural regions central to skill acquisition including dorsolateral prefrontal cortex, posterior parietal cortex, supplementary motor area (SMA), dorsal premotor region (PMd), primary somatosensory cortex, and striatum than their RP counterparts (Lage et al., 2015). In contrast, learning from RP has been reported to involve more extensive activation of parts of the default network often associated with mind-wandering (Wymbs and Grafton, 2009). The importance of planning activity involving some of these regions (i.e., SMA, M1) has been verified using non-invasive stimulation to upregulate activity at these sites during RP leading to improved retention (Kim and Wright, 2020; Kim et al., 2021). Taken together, these data are consistent with the basic tenet of the forgetting-reconstruction account.

It worth noting however that during delayed tests, individuals exposed to IP show reduced recruitment of a number of planning areas enlisted during acquisition (Wymbs and Grafton, 2009). However, the new recruitment profile is coupled with more expansive functional connectivity with (Lin et al., 2013) and heightened cortical excitability at primary motor cortex (M1) (Lin et al., 2011, 2012) up to 72-h following training. Moreover, increased functional connectivity for neural circuits involving SMA and PMd following IP are associated with enhanced learning (Lin et al., 2012, 2018). These outcomes have been interpreted as neural signatures of a more extensive memory network capable of supporting effective retrieval and are in line with the general description detailed in the elaboration perspective.

## Scheduling Practice Variability Can Influence Learning But It Is Less Clear That Variability *per se* Is Sufficient to Advance Skill Memory?

Czyz (2021) was correct in noting that variability of practice is ill-defined in the extant literature. The present commentary focuses on the concept of variability in terms of the intentional practice of skills that are variations of a target skill. This parsimonious definition of practice variability is consistent with that used by Shea and Kohl (1990, 1991) and merely requires accounting for the number of different variations to which a learner is exposed. Importantly, from this perspective, practice variability does not differ when comparing RP and IP formats. The beauty of the CI phenomenon is that *variability, defined in this way, is equated*, thus implicating the *scheduling of the variability* not the variability itself as crucial to securing a learning benefit. This position is counter to the one articulated in the target article and implied by others (Van Rossum, 1990) that IP and RP formats “refer to different degrees of variability” (p. 419).

This latter issue is non-trivial because it raises a fundamental question as to whether we should accept the central position of the target article that the inclusion of variability in a bout of practice is sufficient to aid learning. It should not go unnoticed that almost all studies that include a “variable” practice condition, schedule the presentation of task variations in an interleaved format. As a result, it is impossible to discern if variability *per se* supports learning or, as we claim, it is only when variability is scheduled in a particular manner, that learned is enhanced. This issue is easily resolved if one could demonstrate that a RP condition (or serial practice condition, see Lee and Magill, 1983) can confer a retention benefit beyond constant practice. If exposure to variability *per se* contributes to learning, this should be the case. Going forward, this would seem an important issue to address experimentally.

At this point then it appears that organizing practice variability in an interleaved format exerts a significant role in determining the effectiveness of embedding variability within a bout of practice geared toward acquiring a novel motor skill. As initially detailed by Shea and Kohl (1990, 1991), noted in the target article, and further elaborated herein, learning in this manner results from more extensive recruitment of neural regions known to be central to motor skill acquisition eventually leading to structural and functional neural adaptations that are associated with successful long-term retention (Wright et al., 2016). Unfortunately, at this time, extending such a learning benefit to a more broad-based use of practice variability, awaits additional experimental examination.

## Author Contributions

TK, DW, and WF wrote and edited this manuscript and approved it for publication. All authors contributed to the article and approved the submitted version.

## Conflict of Interest

The authors declare that the research was conducted in the absence of any commercial or financial relationships that could be construed as a potential conflict of interest.

## Publisher's Note

All claims expressed in this article are solely those of the authors and do not necessarily represent those of their affiliated organizations, or those of the publisher, the editors and the reviewers. Any product that may be evaluated in this article, or claim that may be made by its manufacturer, is not guaranteed or endorsed by the publisher.
